# Supplementary material for: GRAF1-dependent endocytotic processes and the Golgi apparatus contribute to previously unrecognized intermediate stages of early ciliogenesis
Source: Nat Commun. 2026 Aug 29;17:9231. doi: 10.1038/s41467-026-76992-5 (PMC13526030; doi:10.1038/s41467-026-76992-5)
Supplement: Supplementary file 1 [file 41467_2026_76992_MOESM1_ESM.pdf]

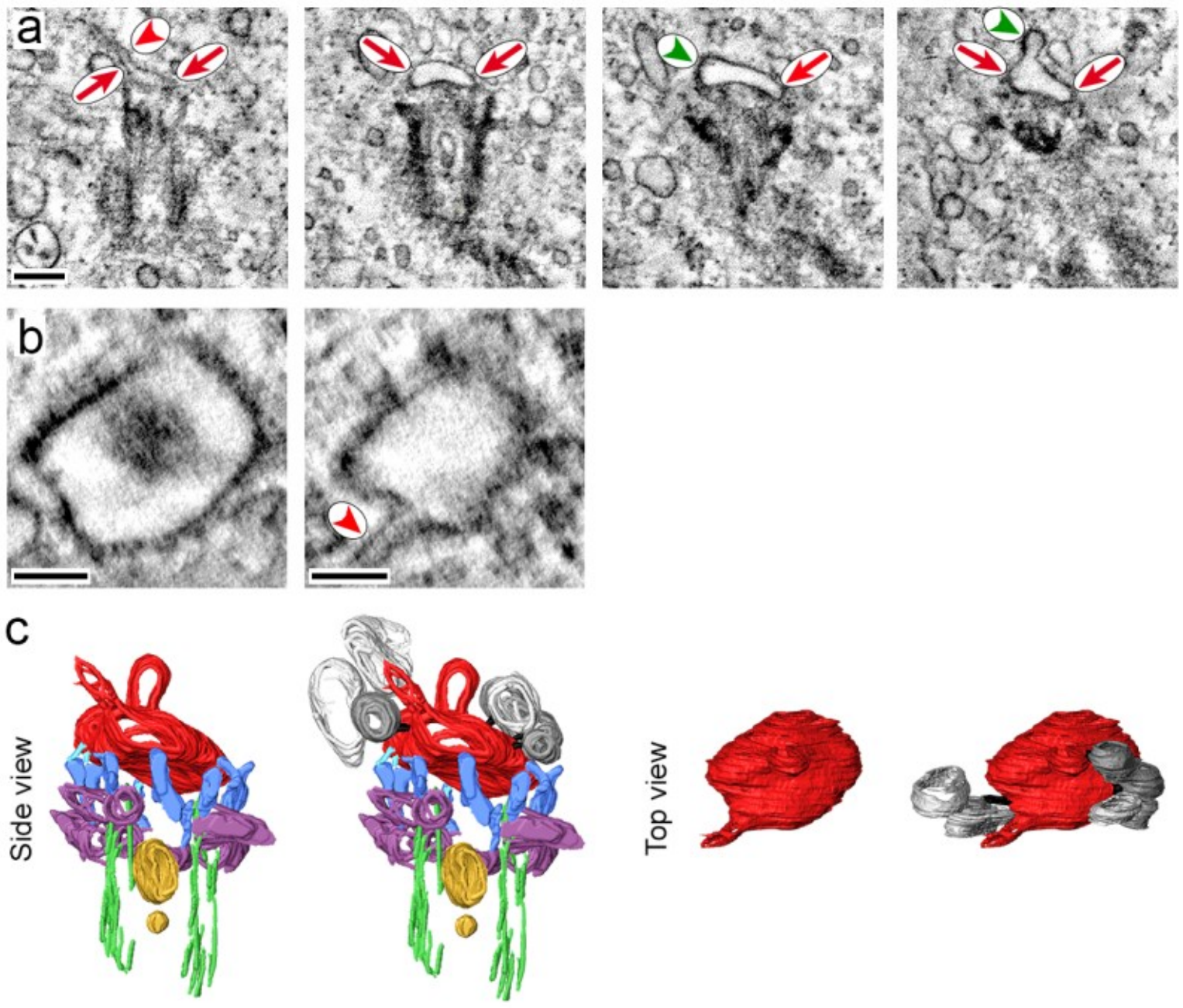

Suppl. Figure 1. Coated buds and attached vesicles at nascent primary cilia. (a) Tomogram of a RPE1 cell stably producing centrin1-EGFP and mCherry-Rab8a in which Rab8a accumulated in a punctate pattern at the centrosome after 3 hours of serum starvation. The slices lie ~55 nm apart, red arrows point to the cap-shaped membrane of the nascent cilium, the red arrow head to a tubular extension. Buds with an electron-dense coat are indicated by green arrow heads. (b) Cross sections of the proximal (left) and distal (right) part of the ciliary membrane structure shown in a. The red arrow head marks a tubular extension. (c) Corresponding segmentation without (left) and with (right) attached vesicles. The dark grey vesicular structures are connected via tethers (shown in black) to the nascent cilium, the light grey vesicular structures directly touch it. Microtubules, green; subdistal appendages, purple; distal appendages, dark blue; tethers between distal appendage and nascent ciliary membrane, light blue; nascent ciliary membrane, red; intracentriolar vesicles, yellow. Bars: 200 nm (a), 100 nm (b).

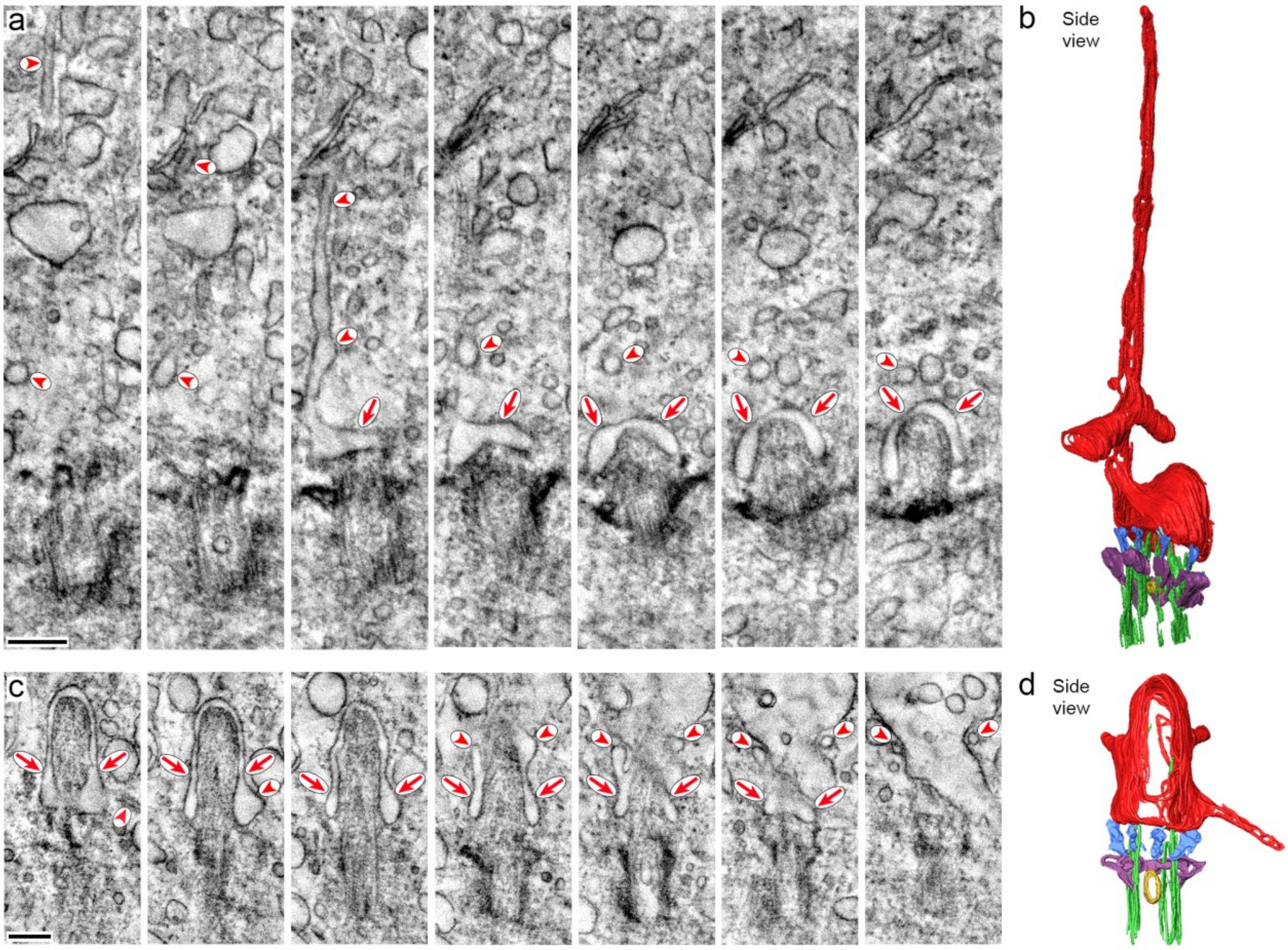

Suppl. Figure 2. Tubular extensions of nascent primary cilia. Two tomograms of RPE1 cells stably producing centrin1-EGFP and mCherry-Rab8a in which Rab8a accumulated in a punctate pattern at the centrosome. Cells were serum-starved for 3 hours (a, b) and for 10 hours (b, d), respectively. (a, c) Seven slices lying ~30 nm apart are shown of each tomogram. Red arrows point to the membrane of the nascent cilium, the red arrow heads to tubular extensions. (b, d) The segmented nascent cilia show that extensions of various lengths exist. Microtubules, green; subdistal appendages, purple; distal appendages, dark blue; tethers between distal appendage and nascent ciliary membrane, light blue; nascent ciliary membrane, red; intracentriolar vesicles, yellow. Bars: 200 nm.

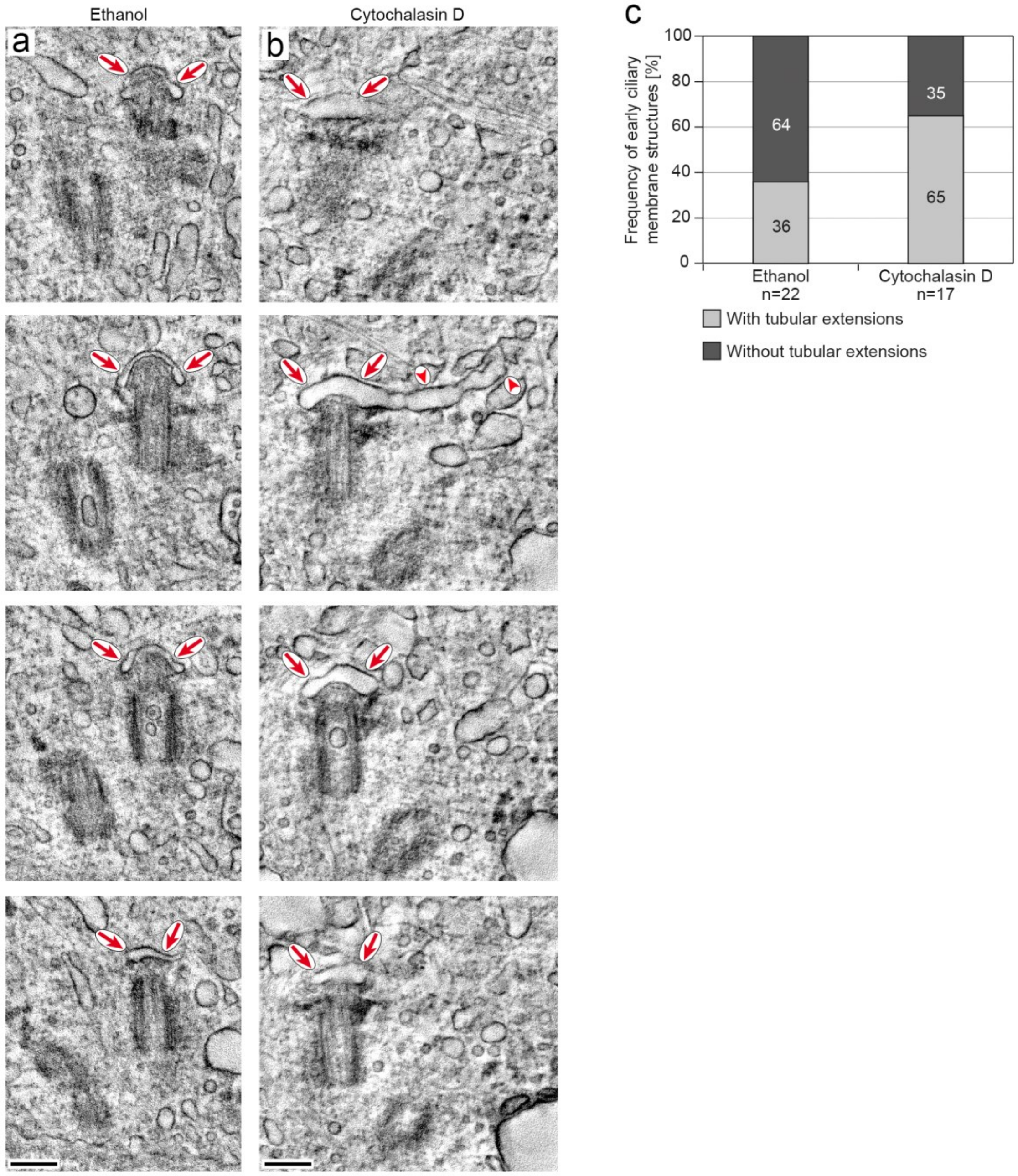

Suppl. Figure 3. Frequency of tubular extensions in cytochalasin D-treated cells. (a, b) RPE1 cells stably producing centrin1-EGFP were serum-starved and simultaneously incubated with ethanol or 50 nM of cytochalasin D for 2 hours. In both tomograms, 4 slices each lying ~50 nm (a) and ~55 nm (b) apart are shown. Red arrows point to the membrane of the nascent cilium, the red arrow heads to a tubular extension. (c) No statistically significant effect on the number of cells with tubular extensions was seen after treatment with cytochalasin D. n = 22 (ethanol-treated cells) and 17 (cytochalasin D-treated cells). n indicates the number of

tomograms. Statistical significance was determined by a two-sided Pearson's chi-square test (panel c). Bars: 200 nm.

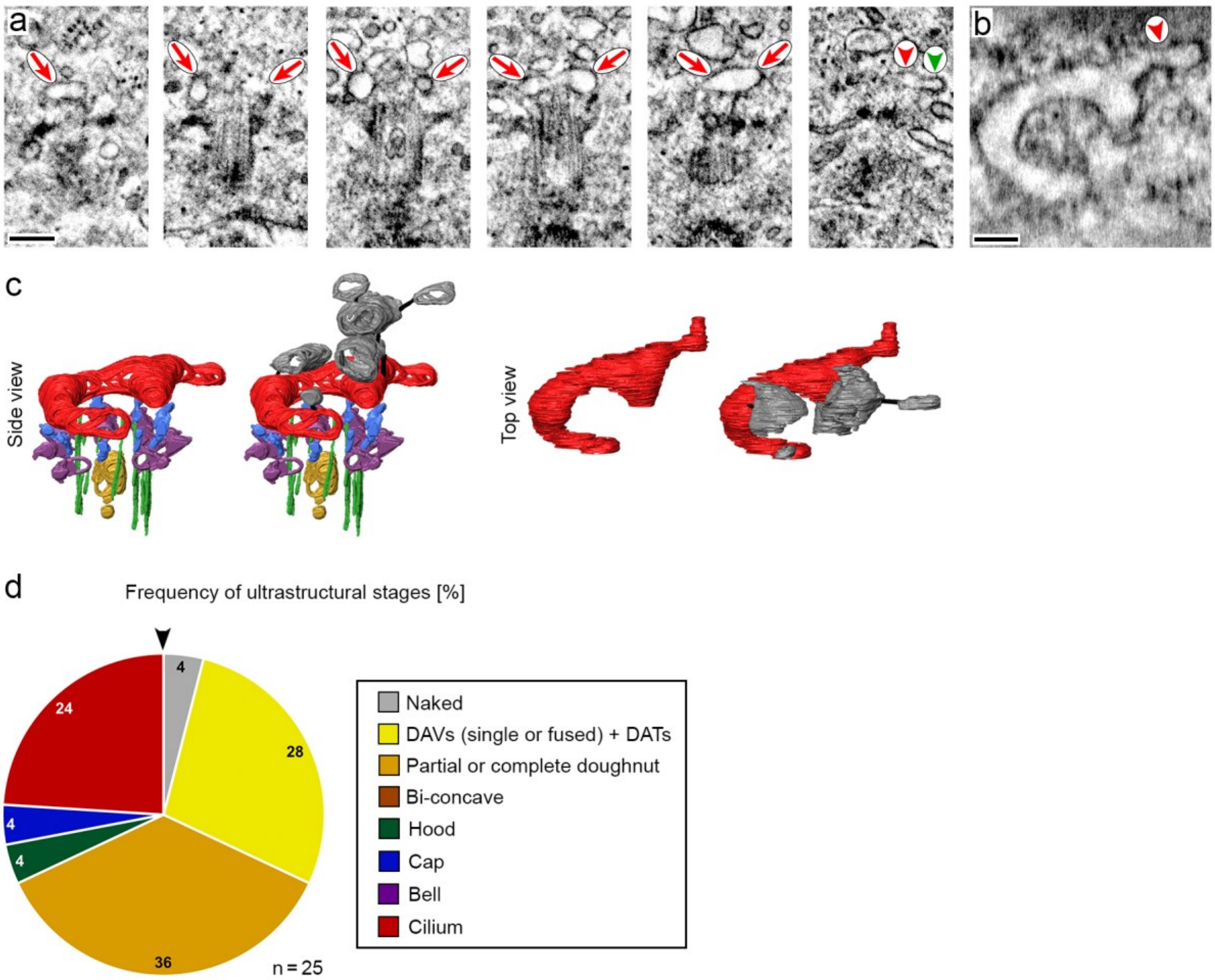

Suppl. Figure 4. Correlative light and electron microscopy of nascent primary cilia in RPE1 cells producing mCherry. RPE1 cells stably producing centrin1-EGFP and transiently transfected with an expression plasmid for mCherry were analyzed after serum deprivation for 3.5 and 10 hours. (a-c) Tomogram of a partial doughnut stage in a cell serum-starved for 10 hours (tomogram identical to that shown in Fig. 2k, l but rotated around the longitudinal axis). Shown are six slices lying ~55 to 60 nm apart (a), a cross section of the partial doughnut-shaped membrane structure (b) and the segmented nascent cilium (c) without (left) and with (right) attached vesicles. Red arrows in the tomograms point to the partial doughnut-shaped membrane structure, the red arrow heads to a tubular extension and the green arrow head to a bud with an electron-dense coat elaborating spikes. The vesicles in dark grey are attached to the partial doughnut-shaped membrane structure and to each other by tethers (shown in black). (d) Pie chart depicting the frequency (in %) of ciliogenesis stages in cells producing mCherry. Early to late stages are portrayed clockwise starting at the arrow head. *n* indicates the number of

tomograms. Microtubules, green; subdistal appendages, purple; distal appendages, dark blue; nascent ciliary membrane, red; intracentriolar vesicles, yellow. Bars: 200 nm (a), 100 nm (b).

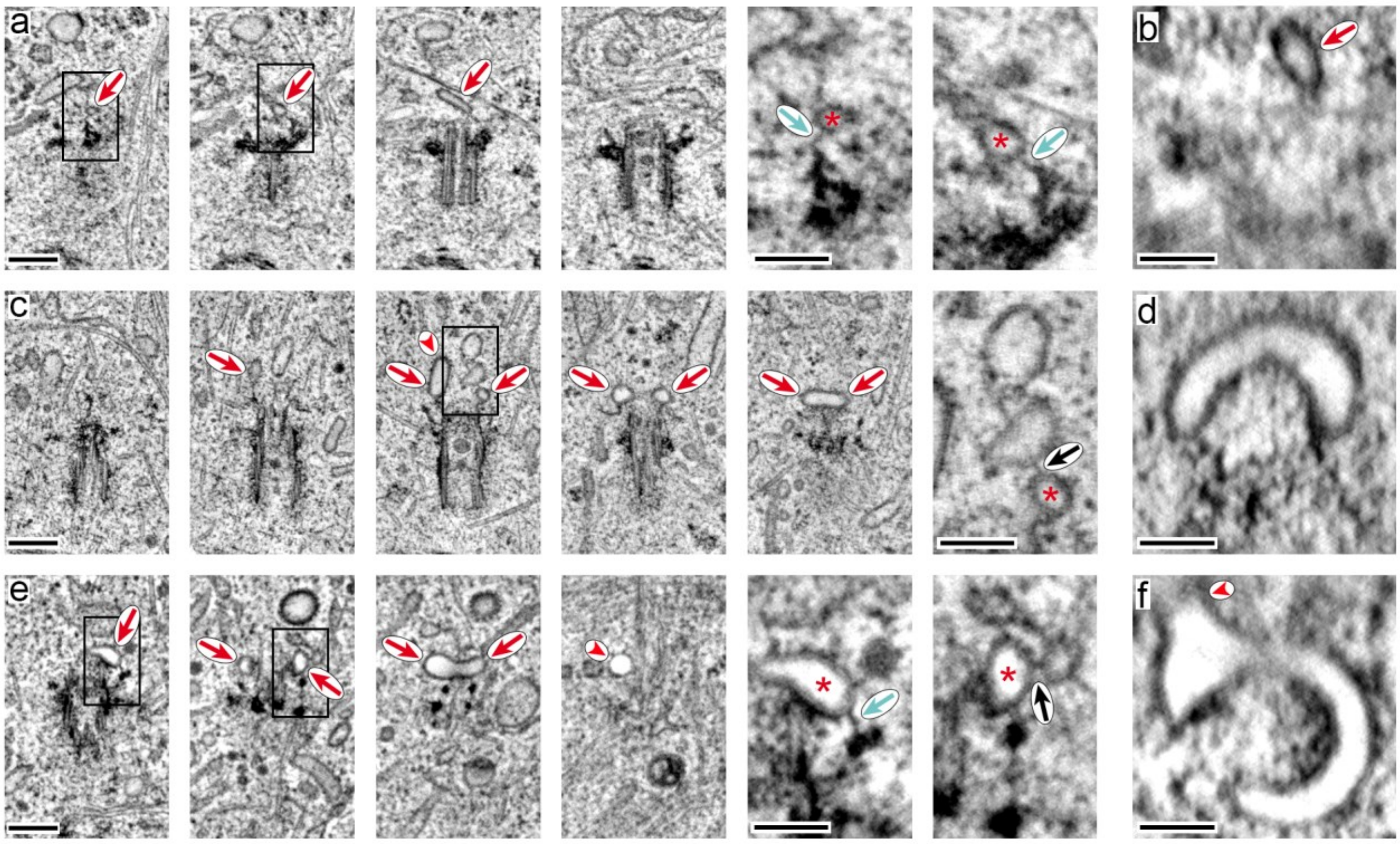

Suppl. Figure 5. Nascent cilia in cells after high-pressure freezing and freeze substitution. Tomograms of RPE1 cells stably producing centrin1-EGFP after 4 hours of serum starvation. (a, c, e) Four slices lying ~34, 28 and 55 nm (a), 5 slices lying ~50, 50, 63 and 50 nm (c) and 4 slices lying ~94, 58 and 50 nm apart are shown of each tomogram. Higher magnifications of the boxed areas are shown in the right-hand panels. (b, d, f) Cross sections of the nascent cilia. Red arrows point to the membrane of the nascent cilium, red asterisks mark the nascent cilium, red arrow heads to tubular extensions, light blue arrows to tethers between distal appendages and the nascent ciliary membrane, and black arrows to tethers between the ciliary membrane and additional vesicles. Bars: 200 nm (overviews in a, c, e), 100 nm (higher magnifications in a, c and e), 100 nm (b, d, f).

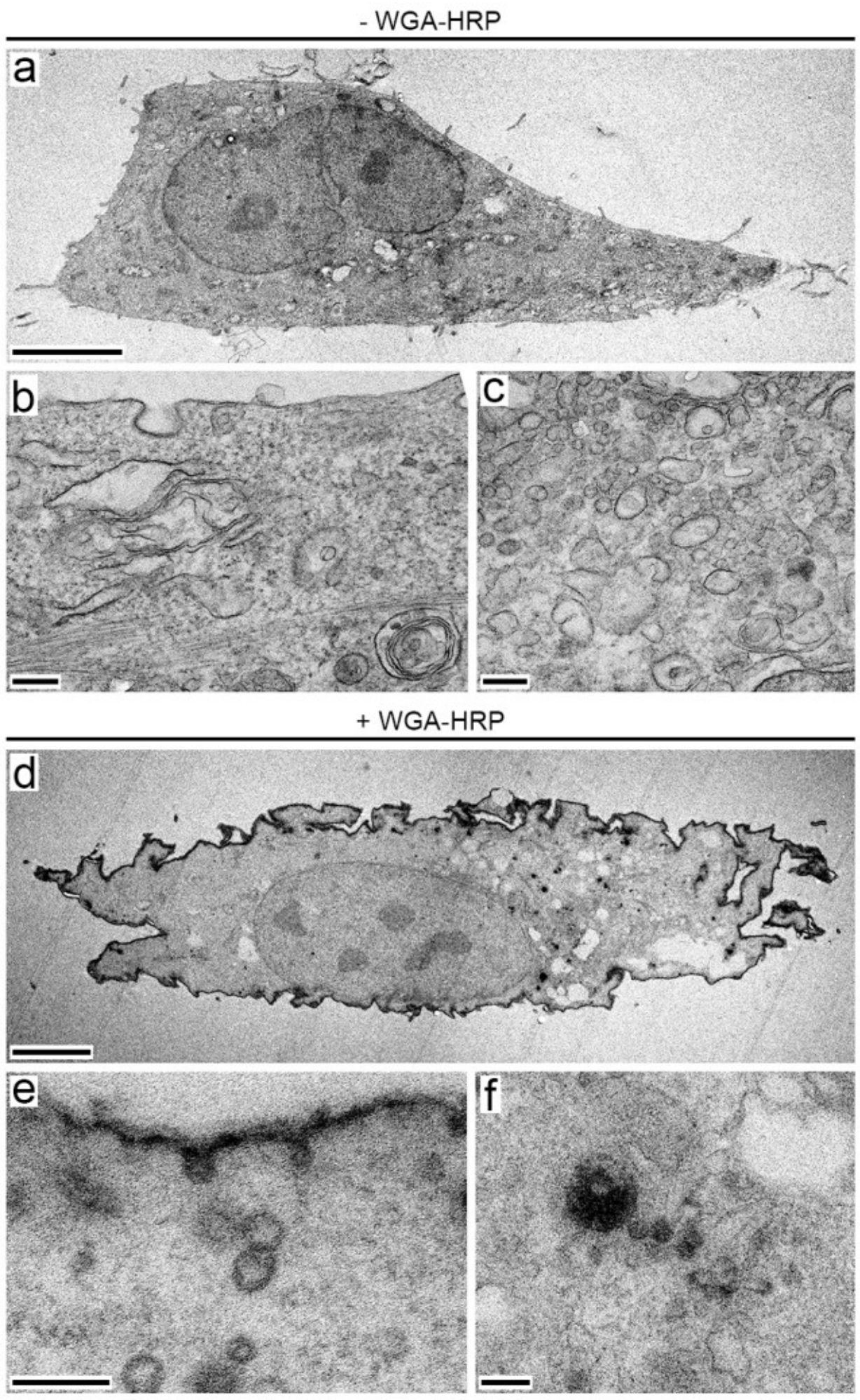

Suppl. Figure 6. WGA-HRP labeling. RPE1 cells stably producing centrin1-EGFP were incubated in the absence (a-c) or presence (d-f) of WGA-HRP. The overviews (a, d) and higher magnifications of plasma membrane regions (b, e) and of the perinuclear cytoplasm (c, f) demonstrates the specific deposition of electron dense material at the plasma membrane and at intracellular vesicles only when WGA-HRP is present. Bars: 5 µm (a, d), 200 nm (b, c, e, f).

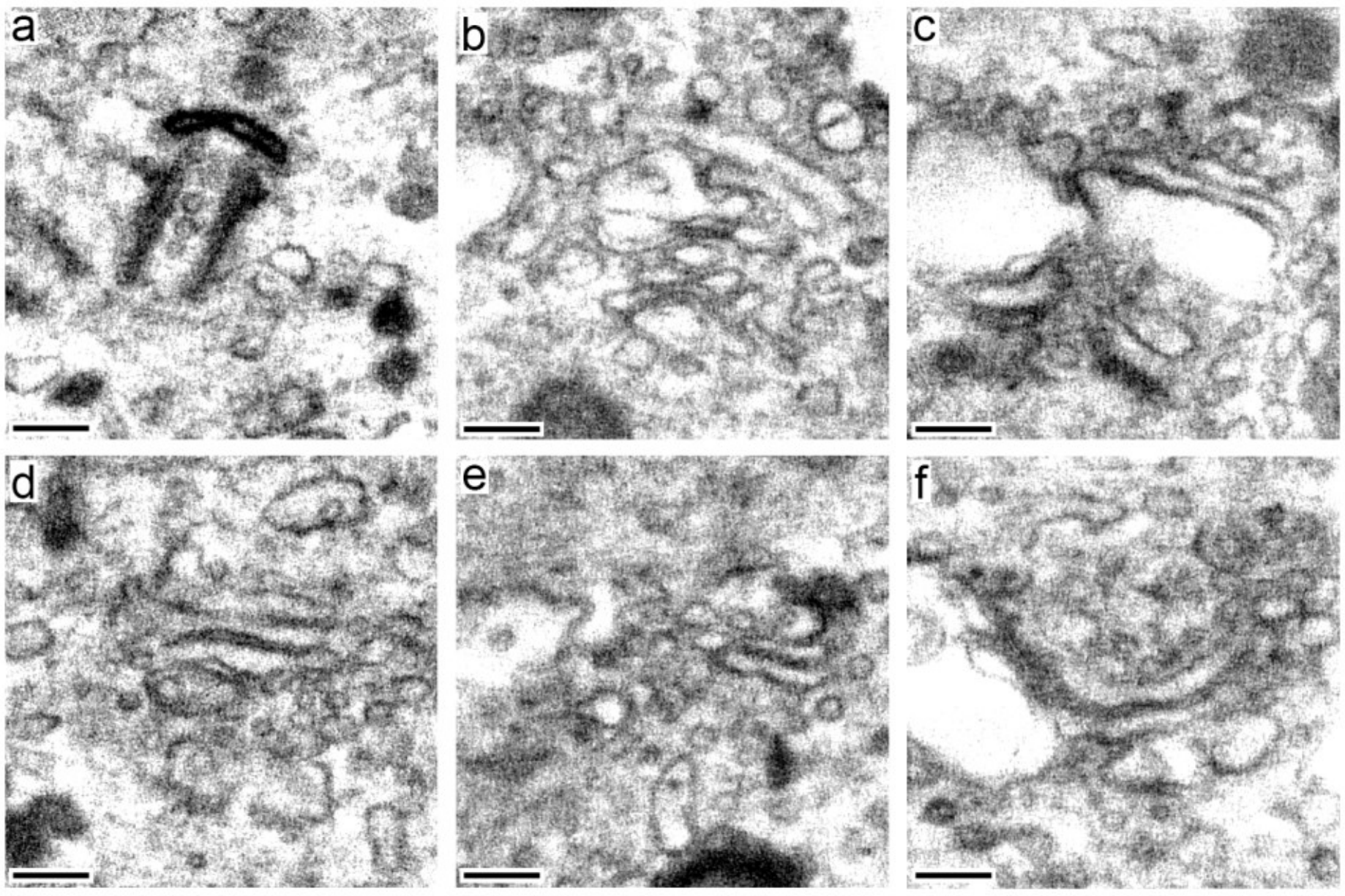

Suppl. Figure 7. Golgi stacks in the vicinity of a WGA-HRP-positive nascent cilium are not labeled with WGA-HRP. RPE1 cells stably producing centrin1-EGFP were serum-starved for 4 hours and exposed to a fusion protein between wheat germ agglutinin (WGA) and horseradish peroxidase (HRP) for the last 15 minutes of serum starvation. An area of 4.5 x 4.5 μm with a thickness of 850 nm was subjected to electron tomography. The WGA-HRP-positive membrane of the nascent cilium already depicted in Fig. 4g, h is shown (a). Five Golgi stacks in the vicinity of the nascent cilium do not show any labeling with WGA-HRP (b-f). Bars: 200 nm.

Supplementary Table 1: Oligonucleotides

Target	Forward primer (5'-3')	Reverse primer (5'-3')
Caveolin1	AAA GAA TTC TGC CAC CAT GTC TGG GGG CAA ATA CGT	TTT GTC GAC TGT ATT TCT TTC TGC AAG TTG ATG
Caveolin1 (DN 1-81)	AAA GAA TTC TGC CAC CAT GGA CGG CAT TTG GAA GGC CA	TTT GTC GAC TGT ATT TCT TTC TGC AAG TTG ATG
Caveolin 1 (P132L)	TCT GGG CAG TTG TAC TAT GCA TTA AGA GCT T	AAG CTC TTA ATG CAT AGT ACA ACT GCC CAG A
EPS15 (DIII)	AAA CTC GAG CTA TGC TCA GCA CCA GCA GCA GTG	TTT GGA TCC TCA TGC TTC TGA TAT CTC AGA
GRAF1	AAA GAA TTC TAT GGG GCT CCC AGC GCT C	TTT GTC GAC TTA GAG GAA CTC CAC GTA ATT CTC
GRAF1 (rescue)	GAA TGC TTC CAG GAC CAC TGA TGA TGT ATC AAT TCC AGA GAA GTT TCA TCA AAG CAG CA	TGCT GCT TTG ATG AAA CTT CTC TGG AAT TGA TAC ATC ATC AGT GGT CCT GGA AGC ATT C
mRuby3	AAA GCT AGC GCT ACC GGT CGC CAC CAT GGT GTC TAA GGG CGA AGA	TTT CTC GAG ACT TGT ACA GCT CGT CCA TGC
Rab5a	AAA GAA TTC TAT GGC TAG TCG AGG CGC AA	TTT GTC GAC TTA GTT ACT ACA ACA CTG ATT CC
Rab11a	AAA GAA TTC TAT GGG CAC CCG CGA CGA C	TTT GTC GAC TTA GAT GTT CTG ACA GCA CTG
