# Supplementary material for: GRAF1-dependent endocytotic processes and the Golgi apparatus contribute to previously unrecognized intermediate stages of early ciliogenesis
Source: Nat Commun. 2026 Aug 29;17:9231. doi: 10.1038/s41467-026-76992-5 (PMC13526030; doi:10.1038/s41467-026-76992-5)
Supplement: Supplementary file 3 [file 41467_2026_76992_MOESM23_ESM.pdf]

nature portfolio

Peer Review File

GRAF1-dependent endocytotic processes and the Golgi apparatus contribute to previously unrecognized intermediate stages of early ciliogenesis

Corresponding Author: Professor Ralph Witzgall

This file contains all reviewer reports in order by version, followed by all author rebuttals in order by version.

Version 0:

Reviewer comments:

Reviewer #1

(Remarks to the Author)
Schmidt and collaborators address the formation and remodelling of the membranes that give rise to the plasma membrane of the primary cilium. The early events that contribute to membrane formation during ciliogenesis are still poorly understood and the authors contribute with an electron microscopy study to this topic.
They show a new doughnut-shaped membrane intermediate that suggests that distal appendage vesicles fuse laterally before filling the space centripetally. The authors also investigate the source of membranes that contribute to ciliogenesis and propose independent roles of the Golgi complex and the CLIC/GEEC endocytic pathway.
In my opinion the study lacks important controls and more evidence needs to be provided to support the author's conclusions. Moreover, while the raw EM data have good quality, the rendering and visualization need substantial improvement.

Main points
1) Segmentations appear quite rough. It looks like the contours of the manual segmentation are not meshed, resulting in holes and pieces of membranes not connected to the rest of the structures. The segmentation data need better meshing and visualization. Moreover, the structures have very complex shapes, which is difficult to appreciate from a few tomogram slices and even worse from the rendering. I would suggest to add supplementary videos with the full tomogram data and rotations/zoom of the segmentations to show the structures in details.
2) Some observations, like the presence of tethers between vesicles or the tubules ending in the cytoplasm, are not very convincing, as they could be sample preparation artifacts. If the authors think this is an important finding, it needs to be confirmed by HPF/FS or complementary methods.
3) The treatments used to inhibit different endocytic pathways are often not very specific. The authors do not see any effect with most of them, concluding that several endocytic pathways are not involved in ciliogenesis. However, it could simply be that the drugs are not working at all in the conditions used. To prove that indeed they have no effect, the authors need to show evidence that those treatments are in fact working to inhibit their respective well know targets at the time and concentrations they used.
4) Involvement of the Golgi in ciliogenesis is not properly demonstrated in my opinion. Data show that a very long BFA treatments (10h) reduces ciliogenesis, but I can imagine that many cellular processes are affected by such a long Golgi inhibition. Can the authors prove that the effect they observed is only due to the Golgi function? Interestingly, the WGA experiment to show that CLIC/GEEC delivery is not impaired by Golgi inhibition is done with 6h BFA incubation. The experimental setups need to be consistent.
5) The role of the tubules emerging from the ciliary membranes is puzzling. The authors mention this structure, but do not show how this is affected by the inhibition of endocytic and Golgi trafficking.
6) To provide a more direct evidence of the fact that the CLIC/GEEC pathway (GRAF1) and the Golgi act on different points of the process, do you achieve an additive effect by inhibiting both?

Minor points:
7) Page 5: ec-CLEM is referred to as "software package", but it is in fact a plug-in of the software package Icy.
8) Page 6/7: speculative language is used to define a sequence of event that cannot be proven in fact, but rather suggested. Eg: "The earliest and most frequent membrane structure attached …" or "the earliest stages at which mCherry-Rab8

localizes" or "newly arriving vesicles (arrows in fig. 3m-p)"
9) Page 7: from the images shown, the presence of coats on the membranes does not prove they are clathrin. Again, this is speculation.
10) Page 11 "The specificity of our approach was corroborated by the finding that many membrane-lined structures in the cytoplasm were not labelled (suppl. Fig. 5)". In my opinion, Figure 5 is better than suppl. Fig. 5 to show this point. The authors should also provide evidence for Golgi not being labelled.
11) Page 11 "Careful 3-dimensional analysis yielded 12 labeled nascent primary cilia that were… . Therefore we conclude that endocytosed membrane material contributes to… " In my opinion, this does not prove the role of endocytosis. Tubule connections could be transiently binding to the ciliary membranes delivering the WGA-labelled material.
12) Page 12 (and elsewhere): the authors mention that their assays have "an effect (without reaching statistical significance". If there is no statistical significance, there is no effect.
13) Page 16 "As we have shown above, Rab8 apparently is required from the partial doughnut stage onwards." The authors show a correlation between the presence of RAb8 and the shape, causality or requirement have never been proven.
14) Page 19 "we would like to point out that not all nascent cilia strictly developed according to this model…" The authors must provide a quantification of the frequency of these other events, as they never appear in any quantification. Also, in my opinion, this suggests that the tendency to divide the process in stages is rather simplistic and arbitrary. It sounds like fusion of the membranes is stochastic.
15) Page 21 "we could not localize the distal ends of these tubules because they lay outside of the tomographed sections". Serial section tomography would be an easy solution to this problem.
16) Page 33 "AMIRA software package (Visage Imaging)" Is it not Amira from Thermo Fisher Scientific?

Reviewer #2

(Remarks to the Author)
In this manuscript, the authors used electron tomography correlated with fluorescence light microscopy to study how membrane remodeling occurs during early ciliogenesis. The results revealed previously unknown details about the membrane process during cilium formation: the initial formation of a doughnut-shaped membrane structure, followed by the closure of its central hole. The manuscript showed that depletion of the endocytosis-associated protein GRAF1 impacts the formation of this doughnut-shaped membrane structure. Additionally, the authors examined the influence of the Golgi apparatus on primary cilium formation. Using detailed 3D ultrastructural data, they proposed a more comprehensive model of early primary cilia development. Overall, this manuscript represents a substantial research effort and offers new ultrastructural insights into the early steps of ciliogenesis. Furthermore, the electron tomographic work demonstrates a successful application of STEM tomography, enabling the authors to work with section thicknesses much greater than those possible with conventional TEM tomography. The manuscript should attract the interest of scientists working in ultrastructural imaging as well as those studying cilium biology. I believe it is a good candidate for publication.

There is some room for improvement before the manuscript is accepted for publication. I have the following comments for the authors to consider:

(1) The manuscript showcases a successful example of electron tomography using STEM imaging instead of traditional TEM imaging. STEM tomography has not been widely adopted in practical applications. It may be useful to include some background on STEM tomography in the introduction, highlighting its advantages for thick specimens.
(2) The manuscript wisely provides background information before presenting the ultrastructural results. For instance, Fig. 1 is used to illustrate the two possible modes of vesicle fusion before referencing data in Figs. 2, 3, and 4 to clarify the fusion process. This approach helps avoid the overly descriptive style common in many structural reports. It could further enhance the manuscript's value by explaining the biological importance of distinguishing between the membrane fusion modes: side-by-side fusion or centripetal fusion, emphasizing why this distinction is crucial.
The authors used the same strategy in the "results" section. However, when the authors introduce speculation beyond established data-based hypotheses, it can sometimes make the results and their interpretation more confusing, potentially leading to misinterpretation by some readers. This issue becomes a notable weakness of the manuscript.
(3) Page 9 Paragraph 2: The second sentence starts a discussion and speculation that "Their function is not known, they may constitute a population of incoming or outgoing vesicles or both." If these vesicles represented mostly incoming vesicles, i.e., vesicles contributing to ciliogenesis, one would expect a larger number in the mother centriole than in the daughter centriole because only the mother centriole will give rise to a primary cilium. Characterization of intracentriolar vesicles." I understand the authors want to provide some background to help interpret the late results. However, the statement is speculative. After all, the paragraph concludes that no direct fusion events are observed, and there is no statistically significant accumulation of these vesicles in either the mother or daughter centriole. The authors end by stating that these intracentriolar vesicles are obscure and of unknown significance. As currently written, this section adds little beyond speculation. It does not add to the overall value of the manuscript. I suggest either removing it or briefly mentioning it in the discussion.
(4) Page 13 Sentence 2: The prior section mentions trends and the presence of endocytosed membrane potentials in primary cilium formation through histochemical-mediated selective staining of endocytosed membrane residues. The current section describes the lack of necessity for clathrin or other endocytotic mediators commonly attributed to cell membrane endocytosis in the context of cilium formation. Could the authors' comment, "If anything, we noticed more cilia when clathrin-mediated endocytosis was inhibited," be spurred on by an increased ability to observe cilia in the light microscope (i.e., increased length, increased presence of cilia which are fully external to the cell)?
(5) Page 14 Sentence 1: "Whereas expression of dominant-negative Rab5a had no effect on ciliogenesis, the expression of

dominant-negative Rab11a reduced the number of primary cilia (Fig. 6i)..." Is this observation truly statistically significant? Is it possible that dominant-negative Rab11a has a cumulative cytotoxic effect similar to that proposed for dynasore and Bafilomycin A1? Different from those in the "Results and Discussion" section, statements in the "Results" section should be more accurate and conclusive.
(6) Pages 30-31: The initial sections indicate that the Correlative Light and Electron Microscopy (CLEM) section is 600 nm thick, but the WGA-HRP section is listed as 900 nm, labeled as "as mentioned earlier." Could the authors explain the reasons for this difference in section thickness?
Since the section thicknesses are much larger than those usually used in ultramicrotomy, the authors should provide more details about their ultramicrotomy process, including the type of the knife (diamond knife? what model and angle?), the cutting angle, and other relevant parameters.
(7) The authors state that at magnifications of 80kx, 120kx, and 200kx, the pixel sizes are 6.69 nm, 2.23 nm, and 1.34 nm. Are these the electron beam spot size and the STEM step size? The details of the STEM imaging should be described accurately.
(8) Page 31, Paragraph 2, Sentence 3: "Micrographs os different magnifications…" should be changed to "micrographs of different magnifications".

Reviewer #3

(Remarks to the Author)
Formation and proper function of the primary cilium in cells have been linked to human diseases called ciliopathies. Since 1962 when the cilia were first observed by electron microscopy, scientists have been able to identify human mutations that are linked to defects on cilia assembly or signaling functions. Using mammalian tissue culture cell models scientists have found that mammalian cells can form cilium by an intracellular and extracellular pathway. On the intracellular ciliogenesis pathway cells will attach vesicles at the distal appendages of the mother centriole that will fuse to form a ciliary vesicle (CV). This CV at the mother centriole will expand and extend part of the cilium axoneme internally before fusing with the plasma membrane to serve as a chemo-and mechanical signaling hub. Some of these early ciliogenesis steps have been identified thanks to the use of ultra-thin section electron microscopy. In addition, molecular machinery related to endosome membrane traffic such as Rab-GTPases and plasma membrane recycling such as EHD1 and Ahi1 have been shown to play a critical role during the formation and dynamics of the CV. The identification of these membrane traffic molecular machinery suggests that endocytic processes are important during the early steps and so the observation of Golgi proximity to the mother centriole. However, the authors pointed out the lack of data demonstrating how the spatial connection between the mother centriole and the Golgi affects ciliogenesis and which compartment supplies the membrane for the intracellular early ciliogenesis events.

To been able to characterize the early ciliogenesis stages with the highest resolution and been able to visualize the different structures of the CV attached to the mother centriole the authors utilize correlative light and electron microscopy (CLEM) with cells stably expressing the centriole marker centrin-EGFP and the CV marker mCherry-Rab8a. With the identification of the centriolar region of the cells the authors then utilized transmission electron microscopy (STEM) tomography to visualize the three-dimensional ultrastructure for the nascent cilia. With this approach the authors were able to identify intermediates ultrastructure during the early stages of ciliogenesis that were not observed before due to the use of ultra-thin 2D electron microscopy. They were able to classify these novel structures such as the partial doughnut enriched with mCherry-Rab8 that by 2D images was previously thought to be individual vesicles attached to the distal appendages.

They used these ultrastructure's classification to characterize the role of membrane endocytosis and Golgi trafficking during ciliogenesis. With the use of WGA-HRP to label plasma membrane internalization, they were able to visualize the accumulation of WGA-HRP vesicles and tubules at the nascent ultrastructure and mature cilia. With the use of pharmacological and genetics manipulations they were able to demonstrate convincingly that the conventional forms of endocytosis (clathrin and caveolin dependent) or the functions of endosomal compartments do not affect the ciliogenesis process. This leads them to identify GRAF1, a protein important for the clathrin-independent carrier (CLIC) and glycosylphosphatidylinositol-anchored proteins (GPI-AP) enriched early endosomal compartment formation (GEEC), to be important for the formation of cilia by decreasing the accumulation of WGA-HRP membrane at the mother centriole. The Golgi role was determined pharmacologically, and the authors observed that BFA treatment cells accumulate more cilia at the distal appendage vesicle or tubule stage (DAV/DAT) than control cells, even due the cilia remained labeled with the WGA-HRP.

They concluded that the Golgi and the GRAF1 endocytic pathway contribute independently to ciliogenesis. Their results and conclusion are a substantial contribution to our understanding of the earliest membrane events and illustrate new regulatory steps during intracellular cilia formation, and these results are well supported by the data they showed. However, I found that the authors need to explore better if the Golgi and GRAF1 are truly independent contributors to ciliogenesis. In addition, I think that the authors need to better describe how the new intermediate structures may help to form a functional cilium and if the cilia that are been form after GRAF1 or Golgi inhibition (40-60%) maybe missing important structural components such as IFT or transition zone components. They made a point regarding the observation of intracentriolar vesicles that they decided not to characterize further since they observed a similar number of these vesicles between the mother and daughter centrioles. I agree that the similar numbers of these vesicles between the centrioles suggest that they may not be specific for cilia formation, but I consider that these vesicles may reflect specific Golgi or endosomes carriers that may show a difference after GRAF1 depletion or Golgi inhibition and deserve further characterization.

Before considering this manuscript for publication Ill like to recommend the authors to perform the following experiments and quantification to clarify the importance of their findings:

1. Figure 1a panel should be merged with figure 2, and panel b should be used on Figure 9 to illustrate which part of the ultrastructure's depend on lateral or centripetal vesicle fusion. This change to figure 2 will make easier to describe what the fluorescence images show and how 3D tomography produces a different view of the Rab8a containing centriole. I also think that figure 4a. b and c should be shown on figure 2 instead, since this data describes the event before the Rab8a accumulation.

2. On figure 5 the authors should show the segmentation of the WGA-HRP membranes as they did on Figure 3 and 4 and highlight cases where the WGA-HRP structures interact with other vesicles. Based on the images they are showing on figure 5 and besides the quantification of the frequency of unlabeled and labeled cilia and I would like the authors to quantify the number and distance of WGA-HRP containing vesicles to the mother centriole. This quantification should also be done in cells depleted of GRAF1 to analyze if part of the phenotypes after GRAF1 depletion is related to vesicle trafficking to the centriole or if the number of carriers is lower. The next figure demonstrates that clathrin dependent endocytosis doesn't affect cilia formation. This result suggests that if they labeled cells with a Tf-HRP cargo the internalize Tf-HRP vesicles should not localize to the mother centriole as WGA-HRP and it will serve as a control that WGA-HRP labeling stimulates a CLIC pathway. They should also label the cells with Galectin3-HRP as a cargo for GEEC that should reproduce what they observed with WGA-HRP.

3. To demonstrate that the Golgi and GRAF1 pathways are independent the authors should analyze the effect of BFA treatment on GRAF1 depleted cells to show that they have an additive effect on the number of ciliated cells as they show with the CEP164 depletion on figure 7b.

4. They showed in figure 8 that the BFA treatment reduced the number of cells with EGFP-Rab8 at the cilia, and this result validated the increase of centriole at the DAV stage they observed in Figure 8k. However, the localization of Rab8a to the centriole was not quantified for GRAF1 depleted cells that also showed an increase in DAV stage cilia when compared to scramble cells.

5. The authors show on Figure 7c and d that the GRAF1 depletion phenotype can be rescued by the expression of EGFP-GRAF1, but they decided not to show where the EGFP-GRAF1 localizes in cells or how the localization changes during ciliogenesis. If the EGFP-GRAF1 localizes to carriers or in proximity to the centriole, they should be able to show by CLEM how the EGFP-GRAF1 localization relates to the mCherry-Rab8a containing centrioles.

6. The RAB11-Rab8 cascade reported by Sahe et al., 2024 demonstrated that overexpression of Rab11 can increase the localization of Rab8 to cilia. In this manuscript they observed the GRAF1 depletion delay the formation of cilia, and if this pathway is independent from the Golgi I would like to know if the over expression of Rab11or Rab8a can rescue the GRAF1 depletion phenotypes. This result will support that the GRAF1 and Golgi pathway contribute independently to cilia formation and that they can compensate for each other. The same paper shows that treatment with Cytochalasin D (CytD) at low dosages can increase the formation of Rab8a tubules, and previously the same treatment was used to induce ciliogenesis to identify actin regulator that promotes cilia formation (Kim et al., 2010). Can the treatment with CytD rescue the GRAF1 depletion or the BFA treatment cilia phenotype and how it may alter the distribution of tubules been form from the partial doughnut or the early cilium structure they observed?

7. A recent paper by Wang et al., 2025 shows that te transition zone component TMEM67 requires core fucosylation by fucosyltransferase 8 (FUT8) to be stabilize and promote ciliogenesis. This N-linked glycosylation will happen at the Golgi compartment. The authors should look at the localization and/or levels of TMEM67 after BFA treatment to determine if it is affected and would explain the importance of Golgi in the localization cilia structural components during ciliogenesis. Are the cilia formed after BFA or GRAF1 depletion treatment contain TMEM67?

8. On supplemental figure 4 the quantification of intracentriolar vesicles demonstrated that expression of mCherry-Rab8a doesn't change significantly the number of intracentriolar vesicles in the mother centriole. However, I would like to know if this quantification was only done with cilia at the partial doughnut stage or if the number of intracentriolar vesicles was different for later stages like early cilium. What effect does the GRAF1 depletion and BFA treatment have on the number of intracentriolar vesicles? Because the tomograph examples on figures 7 and 8 don't look to have clear intracentriolar vesicles as they point out on supplemental figure 4.

9. On the model in Figure 9 I recommend the authors to add where in the model do they think the GRAF1 dependent membrane and the Golgi play their role in order to summarize better their findings.

Version 1:

Reviewer comments:

Reviewer #1

(Remarks to the Author)
The authors have addressed all my points. I would like to thank them for taking my concerns very seriously and for having

conducted a series of control experiments that strengthen their conclusions. I also find the movies of the tomograms very informative and the quality of the STEM data is really impressive.
The answers to the other reviewers are also appropriate, in my opinion.
I believe the manuscript can be published in its current form.

Reviewer #3

(Remarks to the Author)
Reply to rebuttal_comments Reviewer #3
I want to thank Schmidt and the coauthors for addressing my comment to their manuscript. I consider that this revised version addresses the comments and questions I have and their observations and conclusions from the added experiments clarified the importance of the GRAF1 and Golgi trafficking in the formation of the internal ciliary membrane.
I consider that their findings are a substantial contribution to our understanding of the earliest membrane events that regulate intracellular cilia formation and I recommend this manuscript for publication. Congratulations.

(0) Their results and conclusion are a substantial contribution to our understanding of the earliest membrane events and illustrate new regulatory steps during intracellular cilia formation, and these results are well supported by the data they showed.

We are very grateful for the positive comments of the reviewer.

(1) Figure 1a panel should be merged with figure 2, and panel b should be used on Figure 9 to illustrate which part of the ultrastructure's depend on lateral or centripetal vesicle fusion. This change to figure 2 will make easier to describe what the fluorescence images show and how 3D tomography produces a different view of the Rab8a containing centriole. I also think that figure 4a. b and c should be shown on figure 2 instead, since this data describes the event before the Rab8a accumulation.

We have now merged previous Figures 1 and 2.
Appreciate the changes

This request creates somewhat of a difficult situation for us because according to reviewer #2, our „manuscript wisely provides background information before presenting the ultrastructural results. For instance, Fig. 1 is used to illustrate the two possible modes of vesicle fusion before referencing data in Figs. 2, 3, and 4 to clarify the fusion process." We also think that it would be beneficial for readers to leave Figure 1b as is and not to include it into Figure 9 because it serves as the basis to put the subsequent data into the necessary context.

Understand why they prefer to keep it this way.

With respect to moving Figure 4 immediately after Figure 1, we tend to believe that we follow a certain logical order in the way we currently present our results. Rab8a is acknowledged as an early marker of ciliogenesis in the field which is why we started out with tomograms where Rab8a was located at the centrosome. This led to the discovery of the (partial) doughnut stage of ciliogenesis and the filamentous structures connecting the various membrane structures. Based on these data, we subsequently characterized even earlier stages of ciliogenesis.
Therefore we hope for the understanding of this reviewer that we prefer not to change the order of our figures.

Understand.

(2) On figure 5 the authors should show the segmentation of the WGA-HRP membranes as they did on Figure 3 and 4 and highlight cases where the WGA-HRP structures interact with other vesicles. Based on the images they are showing on figure 5 and besides the quantification of the frequency of unlabeled and labeled cilia and I would like the authors to quantify the number and distance of WGA-HRP containing vesicles to the mother centriole. This quantification should also be done in cells depleted of GRAF1 to analyze if part of the phenotypes after GRAF1 depletion is related to vesicle trafficking to the centriole or if the number of carriers is lower. The next figure demonstrates that clathrin dependent endocytosis doesn't affect cilia formation. This result suggests that if they labeled cells with a Tf-HRP cargo the internalize Tf-HRP vesicles should not localize to the mother centriole as WGA-HRP and it will serve as a control that WGA-HRP labeling stimulates a CLIC pathway. They should also label the cells with Galectin3-HRP as a cargo for GEEC that should reproduce what they observed with WGA-HRP.

(i) We have segmented the tomograms containing nascent cilia with WGA-HRP positive membrane structures and highlighted the interactions of those membrane structures with additional vesicles (Fig. 4c, f, i).
Agreed.
(ii) We have determined the number and distance of WGA-HRP positive vesicles to the mother centriole (Fig. 4m, n, o).
Appreciate that the authors analyzed and added these data to the figure.
(iii) We have determined the number and distance of vesicles to the mother centriole after the knock-down of GRAF1. This showed no statistically significant difference in comparison to cells treated with a scrambled siRNA (Fig. 7g, h).

Agreed.

(iv) When we incubated cells with HRP-conjugated transferrin, we observed labeled pits at the plasma membrane but we never observed labeled nascent cilia. Obviously one should be cautious when it comes to the interpretation of negative results but at least by this measure such a result (together with our other data and previously published results) argues against a contribution of clathrin-mediated endocytosis to ciliogenesis. A conjugate between galectin3

and HRP is not commercially available, therefore the respective experiment could not be carried out.
Thank you for disclosing your Tf-HRP observation and understand why it should not be included on the figure.
Understand why the galectin-HRP cannot be perform.

(3) To demonstrate that the Golgi and GRAF1 pathways are independent the authors should analyze the effect of BFA treatment on GRAF1 depleted cells to show that they have an additive effect on the number of ciliated cells as they show with the CEP164 depletion on figure 7b.

The reviewer raises a very interesting point. Indeed, we now demonstrate an additive effect after the inhibition of the Golgi apparatus and the knock-down of GRAF1 (Fig. 8d).
Agreed with the added data.

(4) They showed in figure 8 that the BFA treatment reduced the number of cells with EGFP-Rab8 at the cilia, and this result validated the increase of centriole at the DAV stage they observed in Figure 8k. However, the localization of Rab8a to the centriole was not quantified for GRAF1 depleted cells that also showed an increase in DAV stage cilia when compared to scramble cells.

We have investigated how the location of Rab8a at the centrosome and cilium is affected by the knock-down of GRAF1. Indeed, similar to BFA-treated cells, the depletion of GRAF1 lowers the number of cells where Rab8a is located at the centrosome and cilium (new Fig. 6f).
Agreed.

(5) The authors show on Figure 7c and d that the GRAF1 depletion phenotype can be rescued by the expression of EGFP-GRAF1, but they decided not to show where the EGFP-GRAF1 localizes in cells or how the localization changes during ciliogenesis. If the EGFP-GRAF1 localizes to carriers or in proximity to the centriole, they should be able to show by CLEM how the EGFP-GRAF1 localization relates to the mCherry-Rab8a containing centrioles.

We have transfected cells with an expression plasmid for an mRuby3-GRAF1 fusion protein. Similar to what has been described for an EGFP-GRAF1 fusion protein we observed a punctate distribution in the cytoplasm. We did not, however, detect an altered distribution of GRAF1 after serum starvation. Specifically, we did not detect an accumulation of GRAF1 around the

centrosome. Due to this fact and to the large number of GRAF1 positive structures, which made a precise assignment of vesicles virtually impossible, CLEM was not carried out.

Understand the observation and appreciate the explanation why not to follow up with CLEM.

(6) The RAB11-Rab8 cascade reported by Sahe et al., 2024 demonstrated that overexpression of Rab11 can increase the localization of Rab8 to cilia. In this manuscript they observed the GRAF1 depletion delay the formation of cilia, and if this pathway is independent from the Golgi I would like to know if the over expression of Rab11or Rab8a can rescue the GRAF1 depletion phenotypes. This result will support that the GRAF1 and Golgi pathway contribute independently to cilia formation and that they can compensate for each other. The same paper shows that treatment with Cytochalasin D (CytD) at low dosages can increase the formation of Rab8a tubules, and previously the same treatment was used to induce ciliogenesis to identify actin regulator that promotes cilia formation (Kim et al., 2010). Can the treatment with CytD rescue the GRAF1 depletion or the BFA treatment cilia phenotype and how it may alter the distribution of tubules been form from the partial doughnut or the early cilium structure they observed?

(i) The low number of cilia after the knock-down of GRAF1 could not be rescued by an expression of EGFP-Rab8a (wild-type) and EGFP-Rab11a (wild-type) fusion proteins (Fig. 6g).
Agreed.
(ii) We incubated cells with a knock-down of GRAF1 and cells treated with BFA with cytochalasin D. Indeed, in either case a larger number of ciliated cells was observed compared to control cells (Fig. 6h, Fig. 8g).
Agreed and understand the possible explanation the authors added on the results section for these results.
(iii) We also determined the frequency of tubules extending from nascent cilia after treatment with cytochalasin D. As shown in suppl. Fig. 3, cytochalasin treatment increased the frequency of nascent cilia with tubular extensions.
Agreed.

(7) A recent paper by Wang et al., 2025 shows that te transition zone component TMEM67 requires core fucosylation by fucosyltransferase 8 (FUT8) to be stabilize and promote ciliogenesis. This N-linked glycosylation will happen at the Golgi compartment. The authors should look at the localization and/or levels of TMEM67 after BFA treatment to determine if it

is affected and would explain the importance of Golgi in the localization cilia structural components during ciliogenesis. Are the cilia formed after BFA or GRAF1 depletion treatment contain TMEM67?

We have analyzed the location of TMEM67 after BFA treatment and after the knock-down of GRAF1. In neither case did we observe a decreased number of TMEM67-positive transition zones in the remaining ciliated cells. This is not surprising in the case of the GRAF1 knock-down because we have no evidence that GRAF1 affects the function of the Golgi apparatus. Regarding the treatment with BFA, the situation may be more complex than presented in the Wang paper because FUT8 has also been detected in the endoplasmic reticulum [Glycobiology 36, cwaf087 (2025)].

Thanks for sharing your observation regarding TMEM67 and for sharing that FUT8 may also localize to the endoplasmic reticulum. With this in mind, I understand that analyzing TMEM67 after BFA treatment will be complicated.

(8) On supplemental figure 4 the quantification of intracentriolar vesicles demonstrated that expression of mCherry-Rab8a doesn't change significantly the number of intracentriolar vesicles in the mother centriole. However, I would like to know if this quantification was only done with cilia at the partial doughnut stage or if the number of intracentriolar vesicles was different for later stages like early cilium. What effect does the GRAF1 depletion and BFA treatment have on the number of intracentriolar vesicles? Because the tomograph examples on figures 7 and 8 don't look to have clear intracentriolar vesicles as they point out on supplemental figure 4.

Referee #2 suggested to remove supplemental Figure 4 because our results shed no new light on the function of intracentriolar vesicles and therefore are of unclear significance. We have decided to heed this advice and accordingly would not follow up on the further characterization of these vesicles.
Agreed.

(9) On the model in Figure 9 I recommend the authors to add where in the model do they think the GRAF1 dependent membrane and the Golgi play their role in order to summarize better their findings.
Agreed.
This has been done.

Open Access This Peer Review File is licensed under a Creative Commons Attribution 4.0 International License, which permits use, sharing, adaptation, distribution and reproduction in any medium or format, as long as you give appropriate credit to the original author(s) and the source, provide a link to the Creative Commons license, and indicate if changes were made.
In cases where reviewers are anonymous, credit should be given to 'Anonymous Referee' and the source.
The images or other third party material in this Peer Review File are included in the article's Creative Commons license, unless indicated otherwise in a credit line to the material. If material is not included in the article's Creative Commons license and your intended use is not permitted by statutory regulation or exceeds the permitted use, you will need to obtain permission directly from the copyright holder.

To view a copy of this license, visit https://creativecommons.org/licenses/by/4.0/

Reply to the comments of reviewer #1

(Main point 1) Segmentations appear quite rough. It looks like the contours of the manual segmentation are not meshed, resulting in holes and pieces of membranes not connected to the rest of the structures. The segmentation data need better meshing and visualization. Moreover, the structures have very complex shapes, which is difficult to appreciate from a few tomogram slices and even worse from the rendering. I would suggest to add supplementary videos with the full tomogram data and rotations/zoom of the segmentations to show the structures in details.

Due to the curvature of a given membrane structure it sometimes is very difficult, if not impossible, to identify a membrane with absolute certainty. Moreover, tangential sections through membranes often do not allow the clear identification of those membranes. We decided to play it safe and to err on the side of caution, i.e. if we were not convinced that we recognized a certain structure we did not segment it, which resulted in certain gaps. Notwithstanding this cautious approach, we have now smoothened our data.

We have now generated and uploaded videos which facilitate the visualization of the three-dimensional data.

(Main point 2) Some observations, like the presence of tethers between vesicles or the tubules ending in the cytoplasm, are not very convincing, as they could be sample preparation artifacts. If the authors think this is an important finding, it needs to be confirmed by HPF/FS or complementary methods.

As suggested by the reviewer, we have characterized early ciliogenesis stages by high-pressure freezing and freeze substitution (new suppl. Fig. 5). We have again identified tethers connecting distal appendages and ciliary membrane structures, and tethers connecting already present ciliary membrane structures and additional vesicles. Furthermore, we confirmed the extension of tubules from nascent cilia.

(Main point 3) The treatments used to inhibit different endocytic pathways are often not very specific. The authors do not see any effect with most of them, concluding that several endocytic pathways are not involved in ciliogenesis. However, it could simply be that the drugs are not working at all in the conditions used. To prove that indeed they have no effect, the authors need to show evidence that those treatments are in fact working to inhibit their respective well know targets at the time and concentrations they used.

We have performed the necessary control experiments at 4 and 24 hours of serum deprivation and show that the inhibitors work as used in the ciliogenesis assays. (i) In the case of dynasore, we have used Alexa Fluor 568-conjugated transferrin and observed less uptake, in particular we noticed no perinuclear accumulation of transferrin. (ii) In the case of the vacuolar H^+-ATPase inhibitor bafilomycin A1, we stained cells with LysoTracker Red and demonstrated an almost complete absence of lysosomal staining. (iii) In the case of the phosphatidylinositol (3) kinase inhibitor wortmannin, we used a mCherry-Rab5a fusion protein (a marker of early endosomes) and observed an enlargement of Rab5a-positive structures. (iv) In the case of the Golgi apparatus inhibitors BFA, 30N12 and monensin, we stained for the resident protein GM130 and detected a completely dispersed or at least a structurally altered Golgi apparatus (cf. the fluorescence micrographs below, for simplicity reasons we only show the 4-hour data; the scale bars represent 10 µm).

The assays we used to test the efficacy of methyl-β-cyclodextrin and LY294002 showed no effect at the concentrations we used to inhibit ciliogenesis. Therefore, we decided to remove those data.

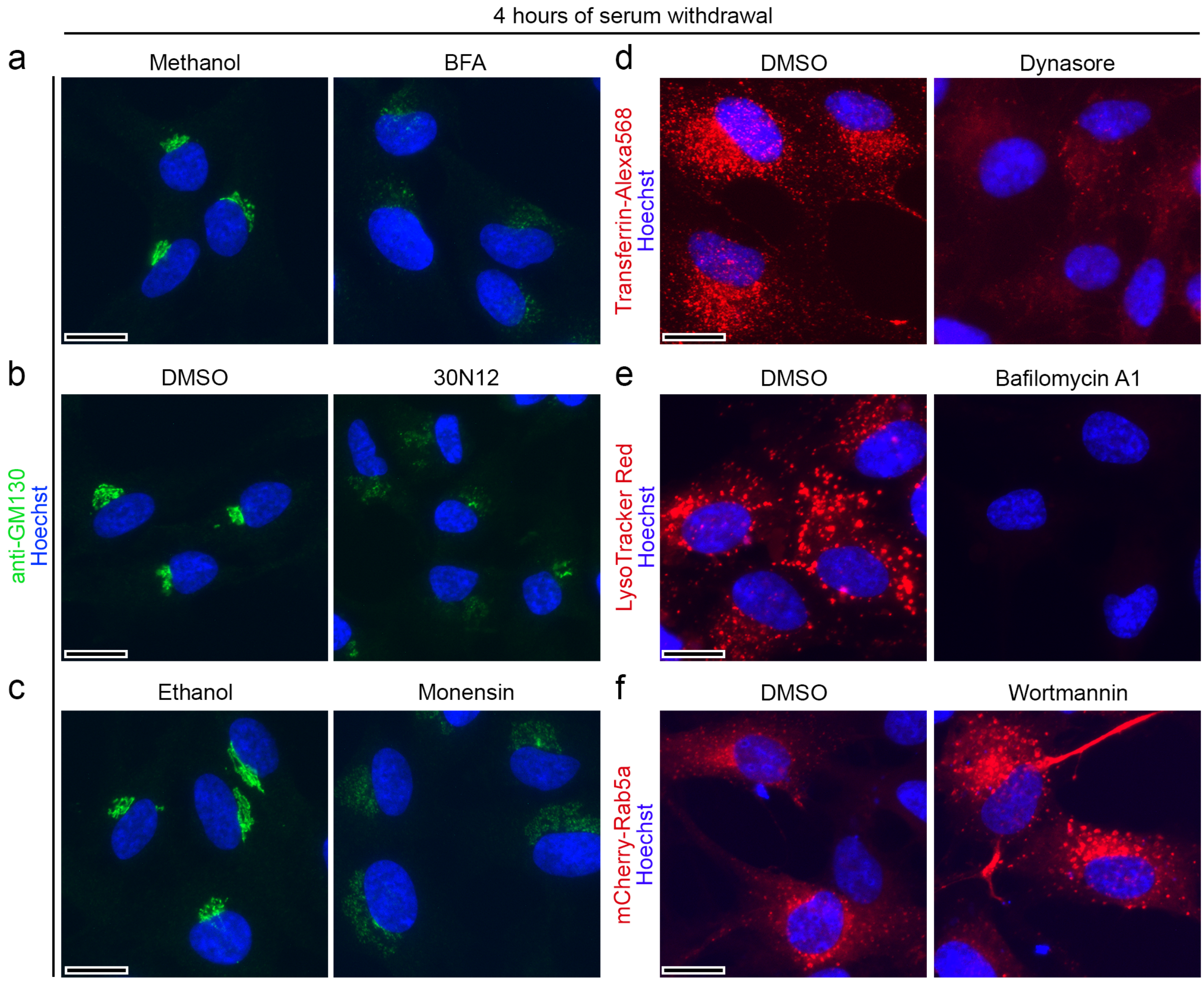

(Main point 4) Involvement of the Golgi in ciliogenesis is not properly demonstrated in my opinion. Data show that a very long BFA treatments (10h) reduces ciliogenesis, but I can imagine that many cellular processes are affected by such a long Golgi inhibition. Can the authors prove that the effect they observed is only due to the Golgi function? Interestingly, the WGA experiment to show that CLIC/GEEC delivery is not impaired by Golgi inhibition is done with 6h BFA incubation. The experimental setups need to be consistent.

The reviewer raises a valid point. We have repeated the experiments underlying Fig. 8a-c using the same setup as for Fig. 8h-n, i.e. 2 hours incubation with the inhibitor in the presence of serum followed by serum deprivation and concomitant further incubation with the inhibitors. We again observe significant differences with the inhibitors. Current Fig. 8a-c shows the new results.

(Main point 5) The role of the tubules emerging from the ciliary membranes is puzzling. The authors mention this structure, but do not show how this is affected by the inhibition of endocytic and Golgi trafficking.

We have now investigated the effect of a GRAF1 knock-down and inhibition of the Golgi apparatus at 4 hours of serum deprivation but did not find a statistically significant effect on the frequency of tubule formation.

(Main point 6) To provide a more direct evidence of the fact that the CLIC/GEEC pathway (GRAF1) and the Golgi act on different points of the process, do you achieve an additive effect by inhibiting both?

The reviewer raises a very interesting point. Indeed, we now demonstrate an additive effect by inhibition of the Golgi apparatus and the simultaneous knock-down of GRAF1 (new Fig. 8d).

(Minor point 7) Page 5: ec-CLEM is referred to as "software package", but it is in fact a plug-in of the software package Icy.

This has been corrected.

(Minor point 8) Page 6/7: speculative language is used to define a sequence of event that cannot be proven in fact, but rather suggested. Eg: "The earliest and most frequent membrane structure attached ..." or "the earliest stages at which mCherry-Rab8 localizes" or "newly arriving vesicles (arrows in fig. 3m-p)"

We have phrased these statements more cautiously.

(Minor point 9) Page 7: from the images shown, the presence of coats on the membranes does not prove they are clathrin. Again, this is speculation.

We fully agree with the reviewer that we did not provide experimental evidence that the coats are clathrin. Accordingly we now mention „vesicles coated with an electron dense material" in the text.

(Minor point 10) Page 11 "The specificity of our approach was corroborated by the finding that many membrane-lined structures in the cytoplasm were not labelled (suppl. Fig. 5)". In my opinion, Figure 5 is better than suppl. Fig. 5 to show this point. The authors should also provide evidence for Golgi not being labelled.

We now refer to both new Figure 4 (previous Fig. 5) and suppl. Figure 6 (previous suppl. Fig. 5) to demonstrate the specificity of labeling membrane structures by WGA-HRP.

We would like to point out that our experimental setup was designed to avoid the retrograde trafficking of WGA-HRP-positive endocytotic vesicles to the Golgi apparatus. It is acknowledged in the literature that an incubation time of 15 minutes is too short for endocytotic vesicles to reach the Golgi apparatus. Nevertheless we have put in a lot of effort and have looked at many tomograms in a number of cells to search for a labeled Golgi apparatus, and were not successful. Of course this does not rule out that a Golgi stack is labeled on certain occasions, but certainly that would not be a regular event. Examples of unlabeled Golgi stacks in the vicinity of a labeled nascent cilium are shown in suppl. Fig. 7.

(Minor point 11) Page 11 "Careful 3-dimensional analysis yielded 12 labeled nascent primary cilia that were… . Therefore we conclude that endocytosed membrane material contributes to… " In my opinion, this does not prove the role of endocytosis. Tubule connections could be transiently binding to the ciliary membranes delivering the WGA-labelled material.

We have acknowledged this possibility a couple of sentences earlier in the Results section („In principle, WGA-HRP might reach the nascent primary cilium not only by endocytosis but also via tubular extensions that connect the developing ciliary membrane compartment with the plasma membrane and thus with the extracellular space") and in the Discussion („In principle, plasma membrane-derived material can reach the ciliary membrane compartment via a direct endocytosis route, by passing through the pericentriolar recycling compartment or through

tubules connecting the plasma membrane with the nascent ciliary membrane."). Nevertheless, we have softened our statement.

(Minor point 12) Page 12 (and elsewhere): the authors mention that their assays have "an effect (without reaching statistical significance". If there is no statistical significance, there is no effect.

This has been corrected.

(Minor point 13) Page 16 "As we have shown above, Rab8 apparently is required from the partial doughnut stage onwards." The authors show a correlation between the presence of RAb8 and the shape, causality or requirement have never been proven.

We have rephrased our argument accordingly.

(Minor point 14) Page 19 "we would like to point out that not all nascent cilia strictly developed according to this model…" The authors must provide a quantification of the frequency of these other events, as they never appear in any quantification. Also, in my opinion, this suggests that the tendency to divide the process in stages is rather simplistic and arbitrary. It sounds like fusion of the membranes is stochastic.

Of the more than 430 tomograms containing centrosomal regions, only 10 exhibited intermediate stages. Due to their rarity they were excluded from our evaluations.

(Minor point 15) Page 21 "we could not localize the distal ends of these tubules because they lay outside of the tomographed sections". Serial section tomography would be an easy solution to this problem.

In principle, serial section tomography sounds like an easy solution. However, you always lose a bit of material through sectioning. Furthermore, even if the relevant structures were present

in the next section it often is challenging to identify and distinguish them e.g. from adjacent vesicles. Therefore in our hands this approach has not fulfilled its promise.

(Minor point 16) Page 33 "AMIRA software package (Visage Imaging)" Is it not Amira from Thermo Fisher Scientific?

AMIRA was provided by Visage Imaging before being taken over by Thermo Fisher Scientific. We now mention ThermoFisher Scientific in the Methods section.

Reply to the comments of reviewer #2

(0) Overall, this manuscript represents a substantial research effort and offers new ultrastructural insights into the early steps of ciliogenesis. Furthermore, the electron tomographic work demonstrates a successful application of STEM tomography, enabling the authors to work with section thicknesses much greater than those possible with conventional TEM tomography. The manuscript should attract the interest of scientists working in ultrastructural imaging as well as those studying cilium biology. I believe it is a good candidate for publication.

We are very grateful for the positive overall assessment of the reviewer.

(1) The manuscript showcases a successful example of electron tomography using STEM imaging instead of traditional TEM imaging. STEM tomography has not been widely adopted in practical applications. It may be useful to include some background on STEM tomography in the introduction, highlighting its advantages for thick specimens.

This has been done in the first paragraph of the Results section.

(2) The manuscript wisely provides background information before presenting the ultrastructural results. For instance, Fig. 1 is used to illustrate the two possible modes of vesicle fusion before referencing data in Figs. 2, 3, and 4 to clarify the fusion process. This approach helps avoid the overly descriptive style common in many structural reports. It could further enhance the manuscript's value by explaining the biological importance of distinguishing between the membrane fusion modes: side-by-side fusion or centripetal fusion, emphasizing why this distinction is crucial.

We now elaborate on this in the respective paragraph.

The authors used the same strategy in the "results" section. However, when the authors introduce speculation beyond established data-based hypotheses, it can sometimes make the

results and their interpretation more confusing, potentially leading to misinterpretation by some readers. This issue becomes a notable weakness of the manuscript.

We have edited our manuscript and have removed sections which may appear too speculative.

(3) Page 9 Paragraph 2: The second sentence starts a discussion and speculation that "Their function is not known, they may constitute a population of incoming or outgoing vesicles or both." If these vesicles represented mostly incoming vesicles, i.e., vesicles contributing to ciliogenesis, one would expect a larger number in the mother centriole than in the daughter centriole because only the mother centriole will give rise to a primary cilium. Characterization of intracentriolar vesicles." I understand the authors want to provide some background to help interpret the late results. However, the statement is speculative. After all, the paragraph concludes that no direct fusion events are observed, and there is no statistically significant accumulation of these vesicles in either the mother or daughter centriole. The authors end by stating that these intracentriolar vesicles are obscure and of unknown significance. As currently written, this section adds little beyond speculation. It does not add to the overall value of the manuscript. I suggest either removing it or briefly mentioning it in the discussion.

We have decided to remove the paragraph in the text and the respective data.

(4) Page 13 Sentence 2: The prior section mentions trends and the presence of endocytosed membrane potentials in primary cilium formation through histochemical-mediated selective staining of endocytosed membrane residues. The current section describes the lack of necessity for clathrin or other endocytotic mediators commonly attributed to cell membrane endocytosis in the context of cilium formation. Could the authors' comment, "If anything, we noticed more cilia when clathrin-mediated endocytosis was inhibited," be spurred on by an increased ability to observe cilia in the light microscope (i.e., increased length, increased presence of cilia which are fully external to the cell)?

We consider the explanation for the detection of more ciliated cells suggested by the reviewer rather unlikely. The ciliary marker employed by us is Arl13b, which is a membrane-associated intracellular protein. In order to detect Arl13b, we have to permeabilize cells with a detergent

no matter whether the (nascent) cilia are still completely contained in the cytoplasm or whether the cilia already extend into the extracellular space. Therefore we believe that it does not matter whether cilia are fully external to the cell or not. Furthermore, by always counter-staining the cells for the centrosomal marker γ-tubulin we made sure that we did not miss even very short cilia.

(5) Page 14 Sentence 1: "Whereas expression of dominant-negative Rab5a had no effect on ciliogenesis, the expression of dominant-negative Rab11a reduced the number of primary cilia (Fig. 6i)..." Is this observation truly statistically significant? Is it possible that dominant-negative Rab11a has a cumulative cytotoxic effect similar to that proposed for dynasore and Bafilomycin A1? Different from those in the "Results and Discussion" section, statements in the "Results" section should be more accurate and conclusive.

There is no question that the effect of dominant-negative Rab11a is statistically significant but at the same time we cannot formally rule out that its expression has a cytotoxic effect. In a way, that is a catch-22 because we need a certain expression level to achieve a dominant-negative effect but that may also lead to unwanted side effects.

We have done our best to phrase the statements in the Results section more accurately and conclusively.

(6) Pages 30-31: The initial sections indicate that the Correlative Light and Electron Microscopy (CLEM) section is 600 nm thick, but the WGA-HRP section is listed as 900 nm, labeled as "as mentioned earlier." Could the authors explain the reasons for this difference in section thickness?

There is a rather trivial explanation for this difference. In the beginning, when we had not optimized a STEM protocol yet, we worked with 600 nm thick sections, only later was it possible for us to use sections with a thickness of 900 nm.

Since the section thicknesses are much larger than those usually used in ultramicrotomy, the authors should provide more details about their ultramicrotomy process, including the type of

the knife (diamond knife? what model and angle?), the cutting angle, and other relevant parameters.

The relevant details are now mentioned in the Methods section.

(7) The authors state that at magnifications of 80kx, 120kx, and 200kx, the pixel sizes are 6.69 nm, 2.23 nm, and 1.34 nm. Are these the electron beam spot size and the STEM step size? The details of the STEM imaging should be described accurately.

This has been done.

(8) Page 31, Paragraph 2, Sentence 3: "Micrographs os different magnifications…" should be changed to "micrographs of different magnifications".

This has been corrected.

Reply to the comments of reviewer #3

(0) Their results and conclusion are a substantial contribution to our understanding of the earliest membrane events and illustrate new regulatory steps during intracellular cilia formation, and these results are well supported by the data they showed.

We are very grateful for the positive comments of the reviewer.

(1) Figure 1a panel should be merged with figure 2, and panel b should be used on Figure 9 to illustrate which part of the ultrastructure's depend on lateral or centripetal vesicle fusion. This change to figure 2 will make easier to describe what the fluorescence images show and how 3D tomography produces a different view of the Rab8a containing centriole. I also think that figure 4a. b and c should be shown on figure 2 instead, since this data describes the event before the Rab8a accumulation.

We have now merged previous Figures 1 and 2.

This request creates somewhat of a difficult situation for us because according to reviewer #2, our „manuscript wisely provides background information before presenting the ultrastructural results. For instance, Fig. 1 is used to illustrate the two possible modes of vesicle fusion before referencing data in Figs. 2, 3, and 4 to clarify the fusion process." We also think that it would be beneficial for readers to leave Figure 1b as is and not to include it into Figure 9 because it serves as the basis to put the subsequent data into the necessary context.

With respect to moving Figure 4 immediately after Figure 1, we tend to believe that we follow a certain logical order in the way we currently present our results. Rab8a is acknowledged as an early marker of ciliogenesis in the field which is why we started out with tomograms where Rab8a was located at the centrosome. This led to the discovery of the (partial) doughnut stage of ciliogenesis and the filamentous structures connecting the various membrane structures. Based on these data, we subsequently characterized even earlier stages of ciliogenesis.

Therefore we hope for the understanding of this reviewer that we prefer not to change the order of our figures.

(2) On figure 5 the authors should show the segmentation of the WGA-HRP membranes as they did on Figure 3 and 4 and highlight cases where the WGA-HRP structures interact with other vesicles. Based on the images they are showing on figure 5 and besides the quantification of the frequency of unlabeled and labeled cilia and I would like the authors to quantify the number and distance of WGA-HRP containing vesicles to the mother centriole. This quantification should also be done in cells depleted of GRAF1 to analyze if part of the phenotypes after GRAF1 depletion is related to vesicle trafficking to the centriole or if the number of carriers is lower. The next figure demonstrates that clathrin dependent endocytosis doesn't affect cilia formation. This result suggests that if they labeled cells with a Tf-HRP cargo the internalize Tf-HRP vesicles should not localize to the mother centriole as WGA-HRP and it will serve as a control that WGA-HRP labeling stimulates a CLIC pathway. They should also label the cells with Galectin3-HRP as a cargo for GEEC that should reproduce what they observed with WGA-HRP.

(i) We have segmented the tomograms containing nascent cilia with WGA-HRP positive membrane structures and highlighted the interactions of those membrane structures with additional vesicles (Fig. 4c, f, i).

(ii) We have determined the number and distance of WGA-HRP positive vesicles to the mother centriole (Fig. 4m, n, o).

(iii) We have determined the number and distance of vesicles to the mother centriole after the knock-down of GRAF1. This showed no statistically significant difference in comparison to cells treated with a scrambled siRNA (Fig. 7g, h).

(iv) When we incubated cells with HRP-conjugated transferrin, we observed labeled pits at the plasma membrane but we never observed labeled nascent cilia. Obviously one should be cautious when it comes to the interpretation of negative results but at least by this measure such a result (together with our other data and previously published results) argues against a contribution of clathrin-mediated endocytosis to ciliogenesis. A conjugate between galectin3

and HRP is not commercially available, therefore the respective experiment could not be carried out.

(3) To demonstrate that the Golgi and GRAF1 pathways are independent the authors should analyze the effect of BFA treatment on GRAF1 depleted cells to show that they have an additive effect on the number of ciliated cells as they show with the CEP164 depletion on figure 7b.

The reviewer raises a very interesting point. Indeed, we now demonstrate an additive effect after the inhibition of the Golgi apparatus and the knock-down of GRAF1 (Fig. 8d).

(4) They showed in figure 8 that the BFA treatment reduced the number of cells with EGFP-Rab8 at the cilia, and this result validated the increase of centriole at the DAV stage they observed in Figure 8k. However, the localization of Rab8a to the centriole was not quantified for GRAF1 depleted cells that also showed an increase in DAV stage cilia when compared to scramble cells.

We have investigated how the location of Rab8a at the centrosome and cilium is affected by the knock-down of GRAF1. Indeed, similar to BFA-treated cells, the depletion of GRAF1 lowers the number of cells where Rab8a is located at the centrosome and cilium (new Fig. 6f).

(5) The authors show on Figure 7c and d that the GRAF1 depletion phenotype can be rescued by the expression of EGFP-GRAF1, but they decided not to show where the EGFP-GRAF1 localizes in cells or how the localization changes during ciliogenesis. If the EGFP-GRAF1 localizes to carriers or in proximity to the centriole, they should be able to show by CLEM how the EGFP-GRAF1 localization relates to the mCherry-Rab8a containing centrioles.

We have transfected cells with an expression plasmid for an mRuby3-GRAF1 fusion protein. Similar to what has been described for an EGFP-GRAF1 fusion protein we observed a punctate distribution in the cytoplasm. We did not, however, detect an altered distribution of GRAF1 after serum starvation. Specifically, we did not detect an accumulation of GRAF1 around the

centrosome. Due to this fact and to the large number of GRAF1 positive structures, which made a precise assignment of vesicles virtually impossible, CLEM was not carried out.

(6) The RAB11-Rab8 cascade reported by Sahe et al., 2024 demonstrated that overexpression of Rab11 can increase the localization of Rab8 to cilia. In this manuscript they observed the GRAF1 depletion delay the formation of cilia, and if this pathway is independent from the Golgi I would like to know if the over expression of Rab11or Rab8a can rescue the GRAF1 depletion phenotypes. This result will support that the GRAF1 and Golgi pathway contribute independently to cilia formation and that they can compensate for each other. The same paper shows that treatment with Cytochalasin D (CytD) at low dosages can increase the formation of Rab8a tubules, and previously the same treatment was used to induce ciliogenesis to identify actin regulator that promotes cilia formation (Kim et al., 2010). Can the treatment with CytD rescue the GRAF1 depletion or the BFA treatment cilia phenotype and how it may alter the distribution of tubules been form from the partial doughnut or the early cilium structure they observed?

(i) The low number of cilia after the knock-down of GRAF1 could not be rescued by an expression of EGFP-Rab8a (wild-type) and EGFP-Rab11a (wild-type) fusion proteins (Fig. 6g).

(ii) We incubated cells with a knock-down of GRAF1 and cells treated with BFA with cytochalasin D. Indeed, in either case a larger number of ciliated cells was observed compared to control cells (Fig. 6h, Fig. 8g).

(iii) We also determined the frequency of tubules extending from nascent cilia after treatment with cytochalasin D. As shown in suppl. Fig. 3, cytochalasin treatment increased the frequency of nascent cilia with tubular extensions.

(7) A recent paper by Wang et al., 2025 shows that te transition zone component TMEM67 requires core fucosylation by fucosyltransferase 8 (FUT8) to be stabilize and promote ciliogenesis. This N-linked glycosylation will happen at the Golgi compartment. The authors should look at the localization and/or levels of TMEM67 after BFA treatment to determine if it

is affected and would explain the importance of Golgi in the localization cilia structural components during ciliogenesis. Are the cilia formed after BFA or GRAF1 depletion treatment contain TMEM67?

We have analyzed the location of TMEM67 after BFA treatment and after the knock-down of GRAF1. In neither case did we observe a decreased number of TMEM67-positive transition zones in the remaining ciliated cells. This is not surprising in the case of the GRAF1 knock-down because we have no evidence that GRAF1 affects the function of the Golgi apparatus. Regarding the treatment with BFA, the situation may be more complex than presented in the Wang paper because FUT8 has also been detected in the endoplasmic reticulum [Glycobiology 36, cwaf087 (2025)].

(8) On supplemental figure 4 the quantification of intracentriolar vesicles demonstrated that expression of mCherry-Rab8a doesn't change significantly the number of intracentriolar vesicles in the mother centriole. However, I would like to know if this quantification was only done with cilia at the partial doughnut stage or if the number of intracentriolar vesicles was different for later stages like early cilium. What effect does the GRAF1 depletion and BFA treatment have on the number of intracentriolar vesicles? Because the tomograph examples on figures 7 and 8 don't look to have clear intracentriolar vesicles as they point out on supplemental figure 4.

Referee #2 suggested to remove supplemental Figure 4 because our results shed no new light on the function of intracentriolar vesicles and therefore are of unclear significance. We have decided to heed this advice and accordingly would not follow up on the further characterization of these vesicles.

(9) On the model in Figure 9 I recommend the authors to add where in the model do they think the GRAF1 dependent membrane and the Golgi play their role in order to summarize better their findings.

This has been done.
