# Supplementary material for: GRAF1-dependent endocytotic processes and the Golgi apparatus contribute to previously unrecognized intermediate stages of early ciliogenesis
Source: Nat Commun. 2026 Aug 29;17:9231. doi: 10.1038/s41467-026-76992-5 (PMC13526030; doi:10.1038/s41467-026-76992-5)
Supplement: Supplementary file 4 [file 41467_2026_76992_MOESM2_ESM.pdf]

Descriptions of Additional Supplementary Files

Supplementary Video 1. Reconstructed tomogram of the partial doughnut stage of cilia formation in a RPE1 cell expressing centrin1-EGFP and mCherry-Rab8a, with mCherry-Rab8a accumulating at the centrosome in a punctate pattern. The movie shows the reconstructed tomogram corresponding to Fig. 1d-g. Scale bar, 200 nm.

Supplementary Video 2. Segmentation of the partial doughnut stage of cilia formation in a RPE1 cell expressing centrin1-EGFP and mCherry-Rab8a, with mCherry-Rab8a accumulating at the centrosome in a punctate pattern. The movie shows the segmented reconstruction of the nascent cilium corresponding to Fig. 1d-g. Microtubules are shown in green, subdistal appendages in purple, distal appendages in dark blue, tethers connecting the distal appendages to the nascent ciliary membrane in light blue, the nascent ciliary membrane in red, and an intracentriolar vesicle in yellow.

Supplementary Video 3. Reconstructed tomogram of the bi-concave stage of cilia formation in a RPE1 cell expressing centrin1-EGFP and mCherry-Rab8a, with mCherry-Rab8a accumulating at the centrosome in a punctate pattern. The movie shows the reconstructed tomogram corresponding to Fig. 2a-c. Scale bar, 200 nm.

Supplementary Video 4. Segmentation of the bi-concave stage of cilia formation in a RPE1 cell expressing centrin1-EGFP and mCherry-Rab8a, with mCherry-Rab8a accumulating at the centrosome in a punctate pattern. The movie shows the segmented reconstruction of the nascent cilium corresponding to Fig. 2a-c. Microtubules are shown in green, subdistal appendages in purple, distal appendages in dark blue, tethers connecting the distal appendages to the nascent ciliary membrane in light blue, the nascent ciliary membrane in red, a vesicle touching the nascent ciliary membrane in light grey, and intracentriolar vesicles in yellow.

Supplementary Video 5. Reconstructed tomogram of the hood stage of cilia formation in a RPE1 cell expressing centrin1-EGFP and mCherry-Rab8a, with mCherry-Rab8a accumulating at the centrosome in a punctate pattern. The movie shows the reconstructed tomogram corresponding to Fig. 2d-f. Scale bar, 200 nm.

Supplementary Video 6. Segmentation of the hood stage of cilia formation in a RPE1 cell expressing centrin1-EGFP and mCherry-Rab8a, with mCherry-Rab8a accumulating at the centrosome in a punctate pattern. The movie shows the segmented reconstruction of the nascent cilium corresponding to Fig. 2d-f. Microtubules are shown in green, subdistal appendages in purple, distal appendages in dark blue, the nascent ciliary membrane in red, a vesicle touching the nascent ciliary membrane in light grey, a vesicle connected to the nascent ciliary via a tether in dark grey, the tether connecting the vesicle to the nascent ciliary membrane in black, and intracentriolar vesicles in yellow.

Supplementary Video 7. Reconstructed tomogram of the cap stage of cilia formation in a RPE1 cell expressing centrin1-EGFP and mCherry-Rab8a, with mCherry-Rab8a accumulating at the centrosome in a punctate pattern. The movie shows the reconstructed tomogram corresponding to Fig. S1. The nascent cilium presents with a tubular extension and two coated buds. Scale bar, 200 nm.

Supplementary Video 8. Reconstructed tomogram of the bell stage of cilia formation in a RPE1 cell expressing centrin1-EGFP and mCherry-Rab8a, with mCherry-Rab8a accumulating at the centrosome in a punctate pattern. The movie shows the reconstructed tomogram corresponding to Fig. 2g-j. Scale bar, 200 nm.

Supplementary Video 9. Segmentation of the bell stage of cilia formation in a RPE1 cell expressing centrin1-EGFP and mCherry-Rab8a, with mCherry-Rab8a accumulating at the centrosome in a punctate pattern. The movie shows the segmented reconstruction of the nascent cilium corresponding to Fig. 2g-j. Microtubules are shown in green, subdistal appendages in purple, distal appendages in dark blue, tethers connecting the distal appendages to the nascent ciliary membrane in light blue, the nascent ciliary membrane in red, vesicles touching the nascent ciliary membrane and each other in light grey, vesicles connected to other vesicles in dark grey, tethers connecting vesicles in black, the plasma membrane in lilac, a tether connecting a vesicle to the plasma membrane in brown, and an intracentriolar vesicle in yellow.

Supplementary Video 10. Reconstructed tomogram of a nascent cilium with tubular extensions in a RPE1 cell expressing centrin1-EGFP and mCherry-Rab8a, with mCherry-Rab8a accumulating at the centrosome in a punctate pattern. The movie shows the reconstructed tomogram corresponding to Fig. S2a, b. Scale bar, 200 nm.

Supplementary Video 11. Segmentation of a nascent cilium with tubular extensions in a RPE1 cell expressing centrin1-EGFP and mCherry-Rab8a, with mCherry-Rab8a accumulating at the centrosome in a punctate pattern. The movie shows the segmented reconstruction of the nascent cilium corresponding to Fig. S2a, b. Microtubules are shown in green, subdistal appendages in purple, distal appendages in dark blue, tethers connecting the distal appendages to the nascent ciliary membrane in light blue, the nascent ciliary membrane in red, and an intracentriolar vesicle in yellow.

Supplementary Video 12. Reconstructed tomogram of distal appendage vesicles in a RPE1 cell expressing centrin1-EGFP and mCherry-Rab8a, with no accumulation of

mCherry-Rab8a at the centrosome. The movie shows the reconstructed tomogram corresponding to Fig. 3b, c. Scale bar, 200 nm.

Supplementary Video 13. Segmentation of distal appendage vesicles in a RPE1 cell expressing centrin1-EGFP and mCherry-Rab8a, with no accumulation of mCherry-Rab8a at the centrosome. The movie shows the segmented reconstruction of the nascent cilium corresponding to Fig. 3b, c. Microtubules are shown in green, subdistal appendages in purple, distal appendages in dark blue, tethers connecting the distal appendages to the nascent ciliary membrane in light blue, the nascent ciliary membrane in red, tubular membrane structure connected via a tether (black) to the nascent ciliary membrane in dark grey, and an intracentriolar vesicle in yellow.

Supplementary Video 14. Reconstructed tomogram of a distal appendage tubule in a RPE1 cell expressing centrin1-EGFP and mCherry-Rab8a, with no accumulation of mCherry-Rab8a at the centrosome. The movie shows the reconstructed tomogram corresponding to Fig. 3d, e. Scale bar, 200 nm.

Supplementary Video 15. Segmentation of a distal appendage tubule in a RPE1 cell expressing centrin1-EGFP and mCherry-Rab8a, with no accumulation of mCherry-Rab8a at the centrosome. The movie shows the segmented reconstruction of the nascent cilium corresponding to Fig. 3d, e. Microtubules are shown in green, subdistal appendages in purple, distal appendages in dark blue, tethers connecting the distal appendages to the nascent ciliary membrane in light blue, the nascent ciliary membrane in red, a vesicle touching the nascent ciliary membrane in light grey, and intracentriolar vesicles in yellow.

Supplementary Video 16. Reconstructed tomogram of the complete doughnut stage of cilia formation in a RPE1 cell expressing centrin1-EGFP and mCherry-Rab8a, with no accumulation of mCherry-Rab8a at the centrosome. The movie shows the reconstructed tomogram corresponding to Fig. 3h-j. Scale bar, 200 nm.

Supplementary Video 17. Segmentation of the complete doughnut stage of cilia formation in a RPE1 cell expressing centrin1-EGFP and mCherry-Rab8a, with no accumulation of mCherry-Rab8a at the centrosome. The movie shows the segmented reconstruction of the nascent cilium corresponding to Fig. 3h-j. Microtubules are shown in green, subdistal appendages in purple, distal appendages in dark blue, a tether connecting a distal appendage to the nascent ciliary membrane in light blue, the nascent ciliary membrane in red, vesicles touching the nascent ciliary membrane and each other in light grey, a vesicle connected to the nascent ciliary membrane via a tether (black) in dark grey, and intracentriolar vesicles in yellow.

Supplementary Video 18. Reconstructed tomogram of the partial doughnut stage of cilia formation in a RPE1 cell expressing centrin1-EGFP and mCherry. The movie shows the reconstructed tomogram corresponding to Fig. S4a-c. The nascent cilium presents with a tubular extension and a coated bud. Scale bar, 200 nm.

Supplementary Video 19. Reconstructed tomogram of a WGA-HRP labeled nascent cilium at the partial doughnut stage of cilia formation in a RPE1 cell expressing centrin1-EGFP. The movie shows the reconstructed tomogram corresponding to Fig. 4d-f. The WGA-HRP-labeled nascent cilium presents with a tubular extension. WGA-HRP-labeled and unlabeled vesicles surround the centrosome. Scale bar, 200 nm.
